# Experimental Detection and Simulation of Terahertz Spectra of Aqueous L-Arginine

**DOI:** 10.3390/bios12111029

**Published:** 2022-11-17

**Authors:** Lei Hou, Junnan Wang, Haiqing Wang, Lei Yang, Wei Shi

**Affiliations:** 1 Department of Physics, Xi’an University of Technology, Xi’an 710048, China; 2 School of Electrical Engineering, Xi’an University of Technology, Xi’an 710048, China; 3 Key Laboratory of Ultrafast Photoelectric Technology and Terahertz Science in Shaanxi, Xi’an University of Technology, Xi’an 710048, China

**Keywords:** terahertz waves, L-arginine, terahertz time-domain spectroscopy system, density functional theory, interaction region indicator

## Abstract

Terahertz (THz) wave is a good candidate for biological sample detection, because vibration and rotation energy levels of biomolecule are in THz band. However, the strong absorption of THz wave by water in biological samples hinders its development. In this paper, a method for direct detection of THz absorption spectra of L-arginine suspension was proposed by using a strong field THz radiation source combined with a polyethylene cell with micrometer thickness in a THz time-domain spectroscopy system. And the THz absorption spectrum of L-arginine solution was simulated by the density functional theory and the simulation result is in good agreement with the experimental results. Finally, the types of chemical bond interaction that cause the absorption peak are identified based on the experimental and simulation results. This work paves a way to investigate the THz absorption spectra and intramolecular interactions of aqueous biological samples.

## 1. Introduction

Terahertz (THz) waves are electromagnetic waves with frequencies between 0.1 and 10 THz and lie between microwave and infrared wave [1]. The single-photon energy of THz waves is only in the order of milli-electron volts, which is comparable to the vibrational rotation energy level of most organic molecules, so THz wave can be used to detect the fingerprint spectra of biological substances without any harmful photoionization [2,3]. Recently, the THz properties of mononuclear ions [4,5,6], small molecules [7,8,9]. In addition, the THz spectroscopy and sensing of biochemical samples have received extensive attention [10,11,12]. Recently, a reflective time-domain polarization spectroscopy system and chiral metasurface sensor to realize high-sensitive quantitative detection of proline, alanine, and tyrosine aqueous solution [13], and realize the proteolysis sensing of bovine serum albumin under the reaction of papain [14]. An all-dielectric metasurface with the functionalized gold nanoparticles was used to qualitatively detect the concentration of HA antigen [15]. THz chiroptical spectroscopy enables the registration and attribution of chiral phonons for microscale and nanoscale crystals of amino acids and peptides [16]. THz spectroscopic detection of amino acids has been significant due to the amino acids play an important role in biomedical, chemical, and food industries [17,18]. Also, THz spectroscopy is an ideal tool to distinguish enantiomers from racemates of amino acids [19,20,21].

Due to the strong absorption of THz wave by water [22,23,24,25], and the slow development of high power THz wave source [26,27,28,29], THz microfluidic detection technology [30,31,32] and THz attenuated total reflection detection technology [33,34,35,36,37], the advancement of THz spectroscopy detection technology for water-containing biological substances is slow. In this paper, we investigated the absorption characteristics of L-arginine aqueous solution in THz band through simulation and experimental detection, because it is a semi-essential amino acid in the body, and its main physiological functions are lowering blood pressure [38,39,40], regulating blood glucose [41], protecting the liver [42], enhancing immunity and limiting tumor growth [43,44,45,46,47], benefit embryo survival and foetal development [48,49,50,51], accelerating wound healing [52,53]. At present, the THz properties of L-arginine are mainly tested with solid samples [54,55], and the computational accuracy in simulating the THz spectra of solid L-arginine still needs to be improved, and the research on microstructure and intramolecular interactions of L-arginine using THz spectroscopy needs to be deepened, and there is no simulation research on its aqueous solution. In the study of the THz characteristics of water-containing samples, some exploratory studies have been carried out to overcome the strong absorption of THz waves by water and thus achieve their THz spectra [56,57,58]. And our group also tested the THz absorption spectra of living Hela cells, aqueous lactose, and aqueous L-arginine [59,60,61] with THz time-domain spectroscopy (THz-TDS) systems. On the basis of previous studies, we measured the THz absorption spectrum of L-arginine aqueous solution with higher accuracy by a THz-TDS system with a strong-field THz source and a polyethylene sample cell in this paper. We also calculated THz spectral properties of aqueous L-arginine by density functional theory (DFT). Combining the experimental and theoretical results, we attributed the molecular vibration modes corresponding to the characteristic peaks, and identified the types and locations of intramolecular interactions in L-arginine aqueous solvent.

## 2. Experiment

### 2.1. Sample Preparation

**Figure 1:**
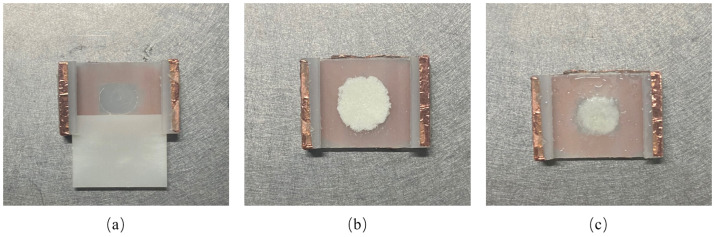
Preparation process of L-Arginine suspension. (**a**) The empty sample cell; (**b**) The sample cell with solid L-arginine power; (**c**) The sample cell with L-arginine suspension.

The L-arginine solid powder used in this paper was provided by the Institute of Modern Physics, Chinese Academy of Sciences, with a purity of more than 98%. Since polyethylene has high transmission properties for THz waves, a polyethylene cell was used to contain L-arginine aqueous solution for THz spectral detection. Figure 1a is the empty sample cell with inner diameter of 7 mm and depth of 100 μm, respectively. In order to eliminate the influence of impurities on the experimental results, the cell was cleaned and dried before testing. After that, solid L-arginine powder was placed into the center of the sample cell, as shown in Figure 1b. Finally, deionized water was sprayed into the L-arginine powder using a small spray bottle to prepare the L-arginine suspension sample, as shown in Figure 1c. The concentration of L-arginine suspension is 0.3 mg/mL.

### 2.2. Experimental Setup

**Figure 2:**
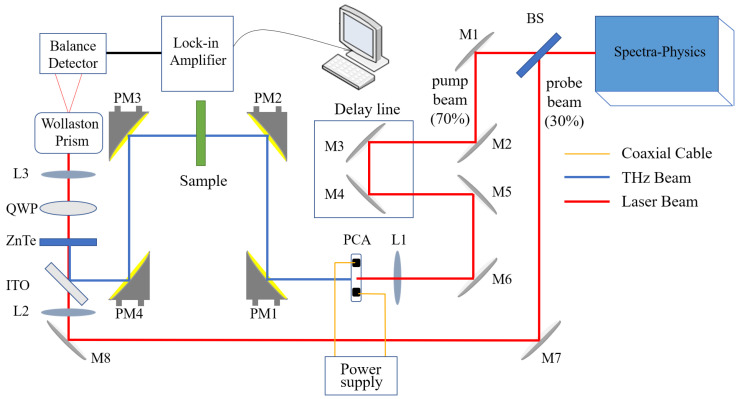
Schematic of THz-TDS system with a sample cell. BS: beam splitter; M: mirror; L: lens; PM: parabolic mirrors; ITO: indium tin oxide; QWP: quarter waveplate; PCA: photoconductive antenna.

The schematic diagram of THz-TDS system for testing the L-arginine suspension is shown in Figure 2. The femtosecond laser (Maitai, Spectra-Physics) was used as light source with central wavelength of 800 nm, pulse width of 70 fs, repetition rate of 1 kHz, and output power of 1 W. The femtosecond laser beam was divided into pump beam and probe beam by a 70/30 beam splitter (BS), the pump beam with 70% of total power is focused on the PCA by the lens (L1) after passing through the delay line to generate THz waves, and the probe beam with 30% of total power is reflected by the mirror (M7, M8) and focused on the ZnTe crystal by the L2. When the focused fs beam illuminated on a biased photoconductive antenna (PCA), the photo-excited surge current was induced and the sub-picosecond THz pulse propagated into the free space. The THz beam was collimated and focused onto the center of the sample cell by two pairs of gold-coated off-axis parabolic mirrors (PM1, PM2). Because the sample is sufficiently thin, so the THz waves can pass through it, and the THz waves carrying the sample’s information was collected by the PM3, PM4, then reflected onto a ZnTe crystal by an indium tin oxide (ITO) film, and the polarization properties of the ZnTe crystal was changed. The probe beam was focused on the ZnTe crystal by the L2, and its polarization state is affected by the ZnTe. When there is no THz electric field, the probe beam after passing through ZnTe is linear polarized. After a quarter wave plate (QWP), its polarization state changes to circular polarization. Wallaston prism (WP) divides the circularly polarized beam into two linearly polarized beams, one is o light and the other is e light, which are respectively received by two photo-diodes of the balance detector (BD). Because the two beams have the same power, the output current of the BD is zero. When a THz pulse electric field and detection pulse is incident on ZnTe crystal at the same time, the probe beam becomes elliptical polarized. Then, the elliptically polarized light passes through the QWP and WP, the intensity of o light and e light becomes different. Finally, the BD loses balance and the differential signal is fed into a lock-in amplifier, and the output current is proportional to the intensity of THz electric field. The time domain waveform of THz wave was obtained by moving the delay line to adjust the relative position of the THz wave and the probe light, and its spectrum can be obtained through Fourier transform.

To reduce the effect of water in L-arginine suspension on the experimental results, deionized water was used as reference. 20 μL deionized water was dropped into the center of the sample cell by a pipette gun and the time domain waveform of the THz wave passing through the water was detected by the THz-TDS system as the reference signal. Afterward, a sample cell containing L-arginine suspension was measured in the THz-TDS system and its THz time domain waveform was obtained. Through the comparison and corresponding treatment of the THz spectra of water and sample, the influence of water is eliminated, and the absorption spectrum of the sample is obtained.

## 3. Theoretical Methods

### 3.1. Model Establishment

**Figure 3:**
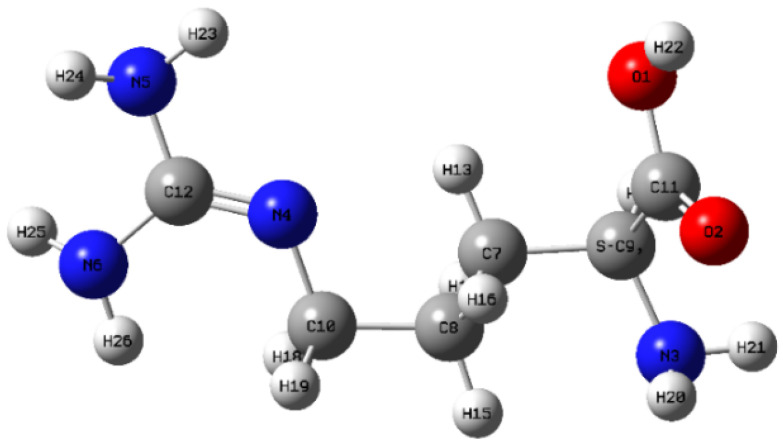
Single-molecule of L-arginine.

In order to investigate the THz spectral properties of L-arginine aqueous solution and understand the relationship between the vibration and rotation characteristics of chemical groups in molecule and their THz spectra, DFT calculation is attractive as an effective tool. The initial structural model of L-arginine was extracted from the PubChem [62] web database, as shown in Figure 3. The hybrid functional B3LYP [63] and the basis set 6-31G(d) were chosen for the simulation. In order to reduce the error caused by B3LYP’s failure to describe the van der Waals force of the calculated system, the dispersion correction term (B3LYP-D3BJ) proposed by Grimme [64] was introduced. Considering that the sample is a kind of aqueous solution, we use an implicit solvent model in the simulation. The model does not specifically describe the specific structure and distribution of solvent molecules near the solute, but regards the solvent environment as a polarizable continuous medium, so the average effect of the solvent can be calculated. In the calculation, the SMD (Solvation Model Based on Density) solvent model from the implicit solvent model [65] was used, the solvent is water, the charge of the system is 0, and the spin multiplicity is 1.

### 3.2. Interaction Region Indicator

In this paper, the Interaction Region Indicator (IRI) [66] was used to analyze the interactions within the L-arginine molecule visually, which is a simple, intuitive and efficient way to identify the type and location of interactions within a molecule. IRI can be defined as follow:

(1) $$\mathrm{IRI}(r)=\frac{\left|\nabla \rho \left(r\right)\right|}{{\left[\rho \left(r\right)\right]}^{a}}$$

where α an adjustable parameter and, α = 1.1 is adopted for standard definition of IRI method, ρ(*r*) is the electron density.

**Figure 4:**
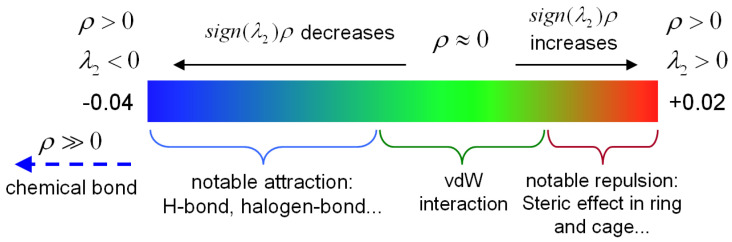
Standard coloring method and chemical explanation of sign(*λ*_2_)ρ on IRI isosurfaces. *λ*_2_ represents the sign of the second largest eigenvalue of the electron density Hessian matrix, and ρ is the electron density. When ρ>0 and $\lambda_{2}<0$, the atoms in the molecule show notable attraction, which corresponds to the blue area. When ρ>0 and $\lambda_{2}>0$, the atoms show notable repulsion, which corresponds to the red area. When ρ≈0, it indicates van der Waals interaction, which corresponds to the green area. When ρ≫0 and $\lambda_{2}<0$, sign(*λ*_2_)ρ smaller than −0.04, and a chemical bond is formed which is indicated by the blue arrow.

The interaction strength and characteristics of different regions are distinguished by projecting the sign(*λ*_2_)ρ onto the IRI equivalent surface, as shown in Figure 4, where, the sign(*λ*_2_) represents the sign of the second largest eigenvalue of the electron density Hessian matrix, which reflects the type of interaction, and ρ(*r*) indicates the strength of the interaction, thus the sign(*λ*_2_)ρ function can be mapped on IRI isosurfaces with different colors to vividly show nature of interaction regions revealed by IRI. The green color indicates the van der Waals interaction region, the red region indicates the presence of certain potential resistance, the blue region indicates significant attraction. The color depth on each color band represents the strength of the interaction.

## 4. Results and Discussion

### 4.1. Experimental and Simulated Spectra of L-Arginine Aqueous Solution

**Figure 5:**
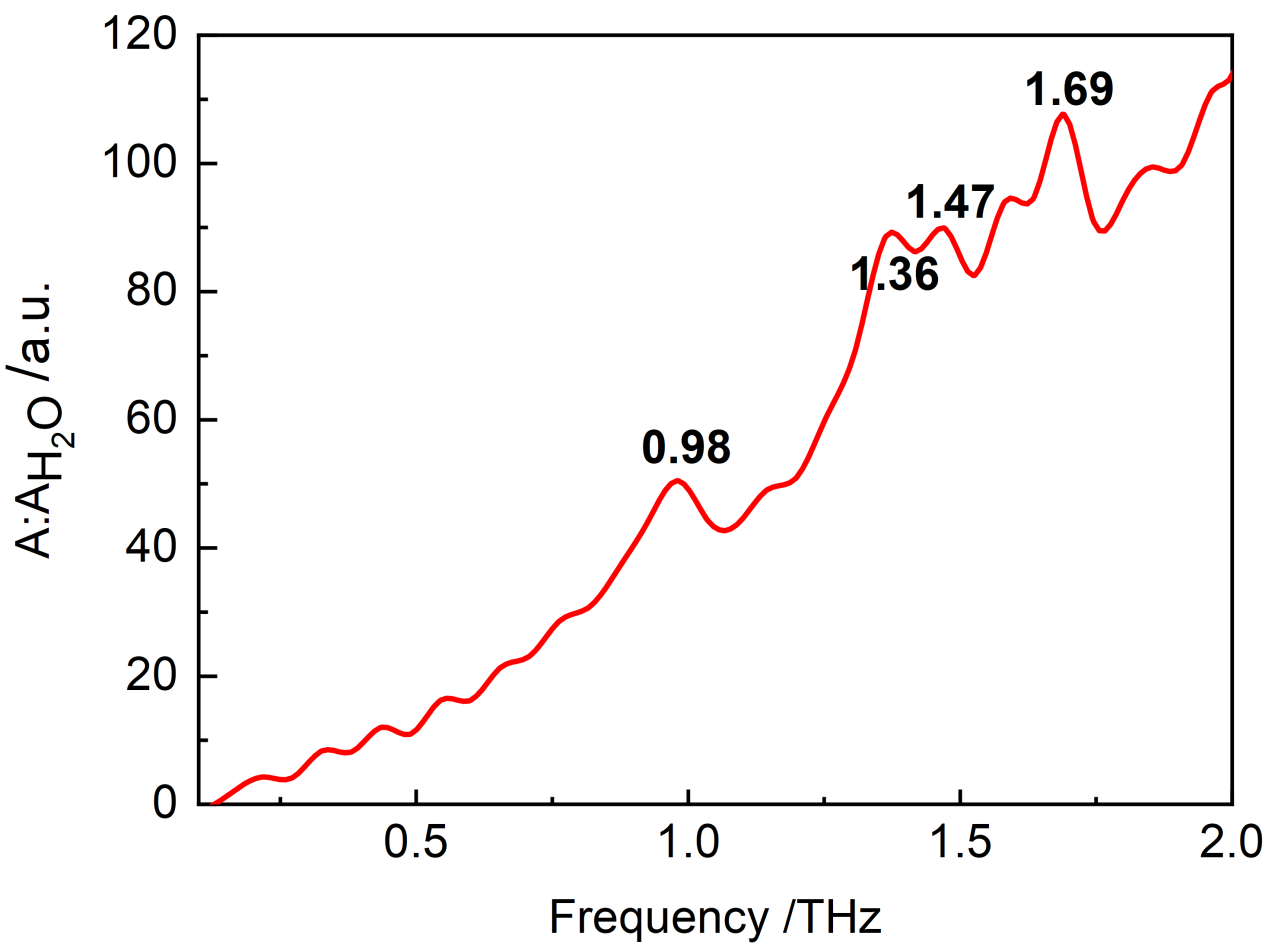
Experimental spectrum of L-arginine suspension, which was obtained by the THz-TDS system with a strong-filed THz source and a polyethylene sample cell. The $A:A_{H_{2}O}$ of the *y*-axis label indicates the ratio of the spectrum of L-arginine suspension to the spectrum when water is used as reference.

By using the experimental method described in 2, the THz spectrum of L-arginine suspension was experimentally measured, as shown in Figure 5. There are four absorption peaks at the frequencies of 0.98, 1.36, 1.47, and 1.69 THz. The absorption peaks at 0.98 and 1.69 THz are more obvious, while the absorption peaks at 1.36 and 1.47 are weaker. The absorption peaks at 0.98, 1.47 and 1.69 THz are consistent with the reported results of solid L-arginine [54,55], as shown in Table 1, while a new absorption peak appears at 1.36 THz, which is caused by the interaction between water molecules and L-arginine molecules. Besides the four obvious absorption peaks, there are some relatively weaker peaks, which may be caused by system noise, or by the interaction between arginine molecules and hydrogen bonds.

**Figure 6:**
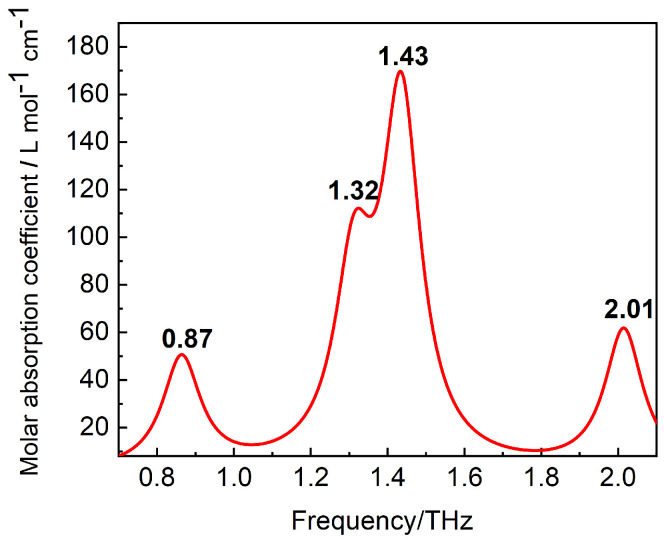
Simulated spectrum of L-arginine suspension in SMD solvent model obtained by B3LYP(D3BJ) and the basis set 6-31G(d), and there are four absorption peaks with the frequencies of 0.87, 1.32, 1.43 and 2.01 THz.

In the calculation, the maximum force, root mean square (RMS) force, maximum displacement and RMS displacement of L-arginine molecular structure meet the requirements of default convergence limit after geometric optimization, and there is no virtual frequency in the frequency calculation results. The calculated THz absorption spectrum of L-arginine solution is shown in Figure 6 by Multiwfn [67] and Origin.

The absorption peaks of simulated spectrum are 0.87, 1.32, 1.43, and 2.01 THz, which respectively correspond to absorption peaks of THz experimental spectra at 0.98, 1.36, 1.47, and 1.69 THz. Compared with the experimental results, the simulation results also have four absorption peaks, and the peak positions are basically consistent. However, the deviation is larger at low frequency and high frequency. In the low frequency range, the vibration of L-arginine tends to the overall vibration, and the single-molecule model used in the simulations fails to take into account the intermolecular interactions, thus leading to a large error of 0.87 THz in the simulated spectrum and 0.98 THz in the experimental spectrum. In the high frequency range, the error between the calculated 2.01 THz and the experimental result of 1.69 THz is larger, mainly because the implicit solvent model used in this simulation affects the potential energy surface of the system to some extent, which will affect the geometric optimization as well as the simulation of the vibration frequencies.

### 4.2. Analysis of Vibrational Modes

**Figure 7:**
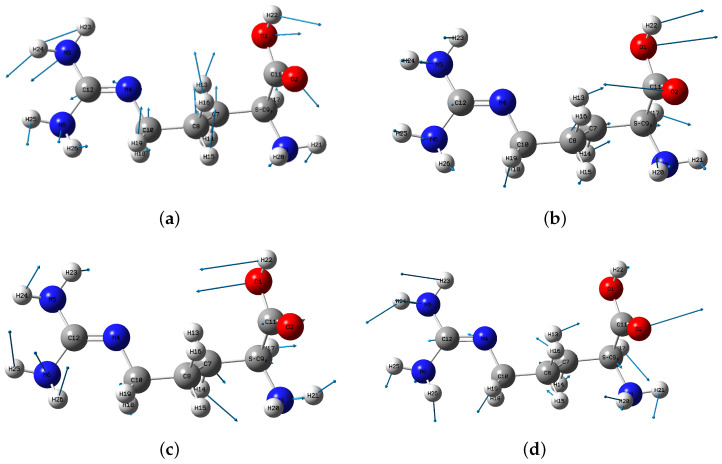
Vibrational modes of L-arginine at (**a**) 0.87 THz; (**b**) 1.32 THz; (**c**) 1.49 THz; (**d**) 2.01 THz obtained by visualization software.

The THz spectrum is related to the molecular structure, and the absorption peaks are intimately bound up with the vibration modes of each group in the molecules. The key word of “freq = intmodes” was added to the frequency calculations when carrying out the theoretical simulation, which can decompose per normal vibration mode into the contribution of each redundant internal coordinate, also contribute to assign the characteristics of vibration mode. To analyze the vibrational modes intuitively, the structural vibration mode of each absorption peak is shown in Figure 7. At the same time, the vibrational modes of L-arginine were attributed to obtain the contribution of major groups and atomic redundant internal coordinates to the normal vibrational modes at different frequencies, and the results are presented in Table 2.

At 0.87 THz, the whole L-arginine molecule drives the guanidine group (C12-N4-N5-H23-H24-N6-H25-H26) and each atom and atomic group on the heterolateral side of the guanidine group to do out-of-plane wagging vibration simultaneously with C10-C8 as the vibration center, as shown in Figure 7a. C9 and C10 and the two methylenes (C8-H15-H16, C7-H13-H14) connected to them make torsional vibration, and their redundant internal coordinate contributions account for 41.9%; the vibration of each atoms and atomic groups with C9 as the center and connected to it accounts for 21.3%. At 1.32 THz, the vibration of L-arginine is mainly a scissoring vibration of atoms and atomic groups on both sides centered on C10-C8, as shown in Figure 7b. The redundant internal coordinate contribution of amino group (N3-H20-H21) centered on the C9 atom and connected to it together with the O2 atom and the hydroxyl group (O1-H22) connected to C11 accounts for 44.5%. At 1.43 THz, the vibration of L-arginine is mainly the twisted vibration of guanidine and the atoms and atomic groups on the opposite side of guanidine with C10-C8 as the center, as shown in Figure 7c. The twist vibration of C7-C8-C10 and the hydrogen atoms connected with it and the vibration of the guanidinium group driven by them accounts for 46.3%; The vibration of atoms and atomic groups with C9 as the center and connected to it accounts for 28.9%. At 2.01 THz, C10-C8 vibration stretching vibration drives all atoms and atomic groups on the opposite side of guanidine group and guanidinium group to do out-of-plane wagging vibration, as shown in Figure 7d. The bending vibration of C10-C8 and the hydrogen atoms connected to it and the guanidine vibration driven by them accounts for 22.4%; The vibration of atoms and atomic groups with C9 as the center and connected to it accounts for 30.2%. The visualization of vibrational mode analysis can explore the origin of the vibration of L-arginine in the THz band.

### 4.3. Visual Analysis of the Interaction with IRI

**Figure 8:**
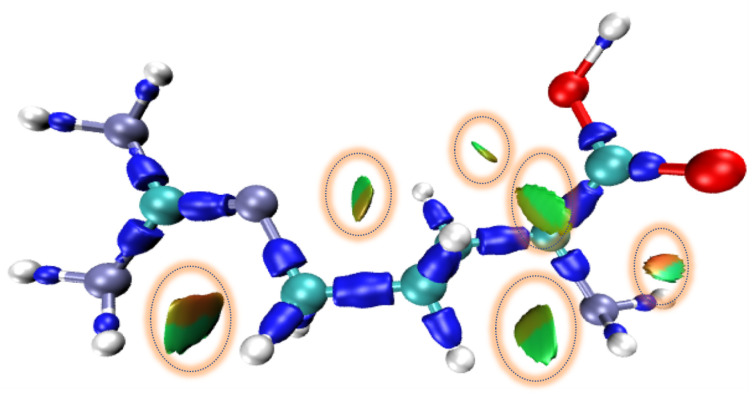
Isosurface map of L-arginine obtained by the VMD, where Carbon, Hydrogen, Oxygen, and Nitrogen atoms are colored in cyan, white grey, red, and slateblue, respectively, and the long dark blue bar indicates the chemical bond. The coloring of this figure is consistent with that of Figure 4.

The chemical bonds and location of weak intramolecular interactions in the system are investigated using the wave function analysis program Multiwfn and the IRI method, and the color filled 3D isosurfaces is drawn using the molecular visualization program VMD. As shown in Figure 8, there are six isosurfaces (marked with orange circles) within the L-arginine molecule, and all of them are dominated by green and red, which indicates that the presence of van der Waals interaction (weak hydrogen bonding) and site-blocking effect in the molecule.

**Figure 9:**
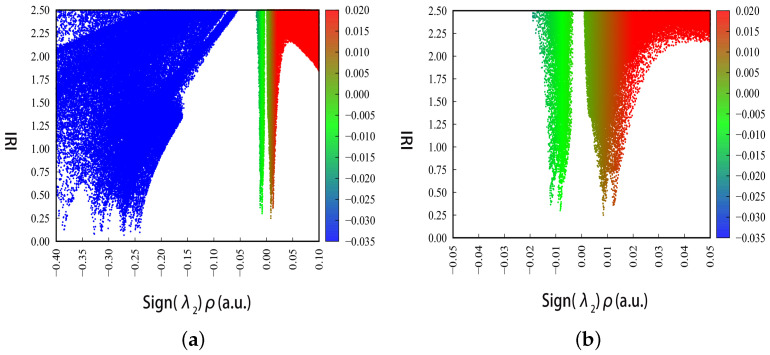
Color scatter maps of L-arginine. (**a**) Color scatter maps with sign(*λ*_2_)ρ from −0.40 to 0.10. (**b**) Color scatter maps with sign(*λ*_2_)ρ from −0.05 to 0.05.

To further quantify the type of weak intramolecular interactions, color scatter maps between IRI and sign(*λ*_2_)ρ of L-arginine were plotted with Multiwfn and Gnuplot software, as shown in Figure 9, and the colors shown in the plot are consistent with those in Figure 4. In Figure 9a, the spikes with the sign(*λ*_2_)ρ value smaller than −0.2 correspond to the chemical bonding within the L-arginine molecule while those with sign(*λ*_2_)ρ between −0.05 and 0.05 indicate weak intermolecular interactions. The region between −0.05 and 0.05 is zoomed in and shown in Figure 9b.

In combination with Figure 8 and Figure 9b, the types and locations of weak intermolecular interactions within the L-arginine molecule can be identified in detail. Both N6-H26 and C10-H19-H18 have green and red isosurfaces, and the red isosurface at N5-C12-N4-C10 corresponds to the spike at 0.008 in Figure 9b, which indicates the site-resistance effect; and the green isosurface indicates the weak hydrogen bonding between the N6 and H18, H19. Four predominantly green and slightly red isosurfaces locate at between N4 and H13, C8-H15 and N3-H20, H16 and O2, O1 and H13, which correspond to the spikes between −0.012 and 0.008 in Figure 9b, where the red isosurface between N4 and H13 indicates the potential resistance effect of N4-C10-C8-C7 and the green one between N4 and H13 represents the weak hydrogen bonding interaction between them. The green isosurface between C8-H15 and N3-H20 indicates a weak hydrogen bonding between N3 and H15, and the slightly red one means there is a weak potential resistance effect of C8-C7-C9-N3. The green isosurface between H16 and O2 indicates a weak hydrogen bonding between them, and the slightly red isosurface indicates a weak site-resistance effect of C8-C7-C9-C11-O2. The green isosurface between O1 and H13 indicates a weak hydrogen bonding between them, and the slightly red isosurface indicates a weak site-resistance effect of H13-C7-C9-C11-O1. A half-red and half-green isosurface exists between O2 and the amino group (N3-H20-H21), the green one indicates a weak hydrogen bonding interaction between O2 and H20, and the red one represents a strong site-resistance effect of O2-C11-C9-N3, corresponding to the 0.012 spike in Figure 9b.

## 5. Conclusions

The THz photon energy is comparable to the vibrational rotation energy level of most organic molecules, so THz wave is suitable for detection the fingerprint spectra of biological substances. However, the strong absorption of THz wave by water in biological samples hinders its development. In order to solve the problem of using THz technology to detect aqueous biological samples and explore the relationship between their characteristic spectra and their molecular structure, we successfully detected the THz spectrum of L-arginine suspension by a THz-TDS system with a high-power THz photoconductive antenna and a sample cell with micrometer size. There are four absorption peaks with the frequencies of 0.98, 1.36, 1.47 and 1.69 THz in the range of 0.1–2.0 THz. DFT was used to simulate the absorption characteristics of aqueous L-arginine, and four absorption peaks with the frequencies of 0.87, 1.32, 1.43, and 2.01 THz were obtained, which are basically consistent with the experimental results. The deviation is caused by the single-molecule model and implicit solvent model approximation in the simulation. The vibrational modes of aqueous L-arginine were analyzed and intramolecular interactions are investigated using the wave function analysis program Multiwfn and the IRI method. The work provides a method to measure the THz absorption spectra and explore weak intramolecular interactions of aqueous biological samples, which is of great significance for people to use THz wave to study life substances and apply them to disease diagnosis in the future.

## Figures and Tables

**Table 1:** The absorption peaks of L-arginine suspension in this experiment and the absorption peaks of L-arginine solid in references.

Sample	Experiment (THz)	Ref. [54] (THz)	Ref. [55] (THz)
L-arginine	0.98	0.99	0.97
	1.36	–	–
	1.47	1.46	1.46
	1.69	2.02	1.68

**Table 2:** Vibrational mode assignment of L-arginine.

Sample	Experiment (THz)	Simulation (THz)	Vibrational Modes
L-arginine	0.98	0.87	C7-C8-C10&C9-C11 (63.2%)
	1.36	1.32	C9-C11 (44.5%)
	1.47	1.43	C7-C8-C10&C9-C11 (75.2%)
	1.69	2.01	C7-C8-C10&C9-C11 (52.6%)

## Data Availability

Not applicable.

## References

[1] Ferguson B., Zhang X.C. (2002). Materials for terahertz science and technology. Nat. Mater..

[2] Liu G., Chang C., Qiao Z., Wu K., Zhu Z., Cui G., Peng W., Tang Y., Li J., Fan C. (2019). Myelin sheath as a dielectric waveguide for signal propagation in the did-infrared to terahertz spectral range. Adv. Funct. Mater..

[3] Jepsen P., Cooke D., Koch M. (2011). Terahertz spectroscopy and imaging—Modern techniques and applications. Laser Photonics Rev..

[4] Heugen U., Schwaab G., Brundermann E., Heyden M., Yu X., Leitner D., Havenith M. (2006). Solute-induced retardation of water dynamics probed directly by terahertz spectroscopy. Proc. Natl. Acad. Sci. USA.

[5] Campo M. (2006). Molecular dynamics simulation of glycine zwitterion in aqueous solution. J. Chem. Phys..

[6] Aikens C., Gordon M. (2006). Incremental solvation of nonionized and zwitterionic glycine. J. Am. Chem. Soc..

[7] Levy Y., Onuchic J. (2006). Water mediation in protein folding and molecular recognition. Annu. Rev. Biophys. Biomol. Struct..

[8] Iftimie R., Tuckerman M. (2006). The molecular origin of the “continuous” infrared absorption in aqueous solutions of acids: A computational approach. Angew. Chem. Int. Ed..

[9] Alonso J., Cocinero E., Lesarri A., Sanz M., Lopez J. (2006). The glycine-water complex. Angew. Chem. Int. Ed..

[10] Gaigeot M., Vuilleumier R., Sprik M., Borgis D. (2005). Infrared spectroscopy of N-methylacetamide revisited by ab initio molecular dynamics simulations. J. Chem. Theory Comput..

[11] Tielrooij K., van der Post S., Hunger J., Bonn M., Bakker H. (2011). Anisotropic water reorientation around ions. J. Phys. Chem. B.

[12] Ramirez R., Lopez-Ciudad T., Kumar P., Marx D. (2004). Quantum corrections to classical time-correlation functions: Hydrogen bonding and anharmonic floppy modes. J. Chem. Phys..

[13] Zhang Z., Zhong C., Fan F., Liu G., Chang S. (2021). Terahertz polarization and chirality sensing for amino acid solution based on chiral metasurface sensor. Sens. Actuators B Chem..

[14] Zhang Z., Zhang T., Fan F., Ji Y., Chang S. (2022). Terahertz polarization sensing of bovine serum albumin proteolysis on curved flexible metasurface. Sens. Actuators A Phys..

[15] Shi W., Fan F., Li S., Zhang Z., Liu H., Wang X., Chang S. (2022). Terahertz immunosensing assisted by functionalized Au NPs based on all-dielectric metasurface. Sens. Actuators B Chem..

[16] Choi W.J., Yano K., Cha M., Colombari F.M., Kim J.Y., Wang Y., Lee S.H., Sun K., Kruger J.M., de Moura A.F. (2022). Chiral phonons in microcrystals and nanofibrils of biomolecules. Nat. Photonics.

[17] Yu B., Zeng F., Yang Y., Xing Q., Chechin A., Xin X., Zeylikovich I., Alfano R. (2004). Torsional Vibrational Modes of Tryptophan Studied by Terahertz Time-Domain Spectroscopy. Biophys. J..

[18] Rungsawang R., Ueno Y., Tomita I., Ajito K. (2006). Angle-dependent terahertz time-domain spectroscopy of amino acid single crystals. J. Phys. Chem. B.

[19] Zhang F., Tominaga K., Hayashi M., Tani M. (2021). A Quantitative Interpretation for the Difference of Terahertz Spectra of dl- and l-Alanine: Origins of Infrared Intensities in Terahertz Spectroscopy. J. Phys. Chem. C.

[20] Yamaguchi M., Miyamaru F., Yamamoto K., Tani M., Hangyo M. (2005). Terahertz absorption spectra of L-, D-, and DL-alanine and their application to determination of enantiometric composition. Appl. Phys. Lett..

[21] Williams M.R., True A.B., Izmaylov A.F., French T.A., Schroeck K., Schmuttenmaer C.A. (2011). Terahertz spectroscopy of enantiopure and racemic polycrystalline valine. Phys. Chem. Chem. Phys..

[22] Litvinov V.M. (2015). Diffusivity of water molecules in amorphous phase of nylon-6 fibers. Macromolecules.

[23] Murthy N.S., Akkapeddi M.K., Orts W.J. (1998). Analysis of lamellar structure in semicrystalline polymers by studying the absorption of water and ethylene glycol in nylons using small-angle neutron scattering. Macromolecules.

[24] Murthy N.S., Stamm M., Sibilia J.P., Krimm S. (1989). Structural changes accompanying hydration in nylon 6. Macromolecules.

[25] Martyna G.J., Klein M.L., Tuckerman M. (1992). Nosé–Hoover chains: The canonical ensemble via continuous dynamics. J. Chem. Phys..

[26] Glyavin M.Y., Denisov G.G., Zapevalov V.E., Koshelev M.A., Tretyakov M.Y., Tsvetkov A.I. (2016). High-power terahertz sources for spectroscopy and material diagnostics. PHYS-USP+.

[27] Li R., Ruan C., Fahad A.K., Zhang C., Li S. (2019). Broadband and high-power terahertz radiation source based on extended interaction klystron. Sci. Rep..

[28] Fokin A., Glyavin M., Golubiatnikov G., Lubyako L., Morozkin M., Movschevich B., Tsvetkov A., Denisov G. (2018). High-power sub-terahertz source with a record frequency stability at up to 1 Hz. Sci. Rep..

[29] Choporova Y., Knyazev B., Pavelyev V. (2022). Holography with high-power CW coherent terahertz source: Optical components, imaging, and applications. Light Adv. Manuf..

[30] Klokkou N.T., Rowe D.J., Bowden B.M., Sessions N.P., West J.J., Wilkinson J.S., Apostolopoulos V. (2022). Structured surface wetting of a PTFE flow-cell for terahertz spectroscopy of proteins. Sens. Actuators B Chem..

[31] Liu L., Pathak R., Cheng L.J., Wang T. (2013). Real-time frequency-domain terahertz sensing and imaging of isopropyl alcohol–water mixtures on a microfluidic chip. Sens. Actuators B Chem..

[32] George P.A., Hui W., Rana F., Hawkins B.G., Smith A.E., Kirby B.J. (2008). Microfluidic devices for terahertz spectroscopy of biomolecules. Opt. Express.

[33] Zang Z., Li Z., Lu X., Liang J., Wang J., Cui H.L., Yan S. (2021). Terahertz spectroscopy for quantification of free water and bound water in leaf. Comput. Electron. Agric..

[34] Lajevardipour A., Vilagosh Z., Appadoo D., Davis J., Juodkazis S., Wood A. (2021). Spectroscopy of excised skin patches exposed to THz and far-IR radiation. Biomed. Opt. Express.

[35] Liao Y., Zhang M., Tang M., Chen L., Li X., Liu Z., Wang H. (2022). Label-free study on the effect of a bioactive constituent on glioma cells in vitro using terahertz ATR spectroscopy. Biomed. Opt. Express.

[36] Mu N., Yang C., Xu D., Wang S., Ma K., Lai Y., Guo P., Zhang S., Wang Y., Feng H. (2022). Molecular pathological recognition of freshly excised human glioma using terahertz ATR spectroscopy. Biomed. Opt. Express.

[37] Wang Y., Jiang Z., Xu D., Chen T., Chen B., Wang S., Mu N., Feng H., Yao J. (2019). Study of the dielectric characteristics of living glial-like cells using terahertz ATR spectroscopy. Biomed. Opt. Express.

[38] Kibe R., Kurihara S., Sakai Y., Suzuki H., Ooga T., Sawaki E., Muramatsu K., Nakamura A., Yamashita A., Kitada Y. (2014). Upregulation of colonic luminal polyamines produced by intestinal microbiota delays senescence in mice. Sci. Rep..

[39] Gokce N. (2004). L-Arginine and hypertension. J. Nutr..

[40] Andrew P.J., Mayer B. (1999). Enzymatic function of nitric oxide synthases. Cardiovasc. Res..

[41] Hu S., Han M., Rezaei A., Li D., Wu G., Ma X. (2017). L-Arginine modulates glucose and lipid metabolism in obesity and diabetes. Curr. Protein Pept. Sci..

[42] Chattopadhyay P., Verma N., Verma A., Kamboj T., Khan N.A., Wahi A.K. (2008). L-arginine protects from pringle manoeuvere of ischemia-reperfusion induced liver injury. Biol. Pharm. Bull..

[43] Canale F.P., Basso C., Antonini G., Perotti M., Li N., Sokolovska A., Neumann J., James M.J., Geiger S., Jin W. (2021). Metabolic modulation of tumours with engineered bacteria for immunotherapy. Nature.

[44] Rodríguez P.C., Ochoa A.C. (2008). Arginine regulation by myeloid derived suppressor cells and tolerance in cancer: Mechanisms and therapeutic perspectives. Immunol. Rev..

[45] Bronte V., Zanovello P. (2005). Regulation of immune responses by L-arginine metabolism. Nat. Rev. Immunol..

[46] Geiger R., Rieckmann J.C., Wolf T., Basso C., Feng Y., Fuhrer T., Kogadeeva M., Picotti P., Meissner F., Mann M. (2016). L-Arginine Modulates T Cell Metabolism and Enhances Survival and Anti-tumor Activity. Cell.

[47] Martí i Líndez A.A., Dunand-Sauthier I., Conti M., Gobet F., Núñez N., Hannich J.T., Riezman H., Geiger R., Piersigilli A., Hahn K. (2020). Mitochondrial arginase-2 is a cell-autonomous regulator of CD8^+^ T cell function and antitumor efficacy. JCI Insight.

[48] Palencia J.Y.P., Lemes M.A.G., Garbossa C.A.P., Abreu M.L.T., Pereira L.J., Zangeronimo M.G. (2018). Arginine for gestating sows and foetal development: A systematic review. J. Anim. Physiol. Anim. Nutr..

[49] El-Sherbiny H.R., Samir H., El-Shalofy A.S., Abdelnaby E.A. (2022). Exogenous L-arginine administration improves uterine vascular perfusion, uteroplacental thickness, steroid concentrations and nitric oxide levels in pregnant buffaloes under subtropical conditions. Reprod. Domest. Anim..

[50] Li X., Bazer F.W., Johnson G.A., Burghardt R.C., Erikson D.W., Frank J.W., Spencer T.E., Shinzato I., Wu G. (2010). Dietary Supplementation with 0.8% L-Arginine between Days 0 and 25 of Gestation Reduces Litter Size in Gilts. J. Nutr..

[51] Zhang H., Sun L., Wang Z., Deng M., Nie H., Zhang G., Ma T., Wang F. (2016). N-carbamylglutamate and L-arginine improved maternal and placental development in underfed ewes. Reproduction.

[52] Stechmiller J.K., Childress B., Cowan L. (2005). Arginine supplementation and wound healing. Nutr. Clin. Pract..

[53] Witte M.B., Barbul A. (2003). Arginine physiology and its implication for wound healing. Wound Repair Regen..

[54] Li T., Yu Q., Zhang L., Jiang L. (2020). Terahertz spectroscopy of amino acid crystals based on dispersion-correction functional theory. Spectrosc. Lett..

[55] Ye P., Wang G., Yang Y., Meng Q., Wang J., Su B., Zhang C. (2021). Terahertz absorption properties of two solid amino acids and their aqueous solutions. Int. J. Opt..

[56] Exter M., Fattinger C., Grischkowsky D. (1989). Terahertz time-domain spectroscopy of water vapor. Opt. Lett..

[57] Grognot M., Gallot G. (2015). Quantitative measurement of permeabilization of living cells by terahertz attenuated total reflection. Appl. Phys. Lett..

[58] Shih K., Pitchappa P., Jin L., Chen C.H., Singh R., Lee C. (2018). Nanofluidic terahertz metasensor for sensing in aqueous environment. Appl. Phys. Lett..

[59] Wang H., Shi W., Hou L., Wang Z., Wu M., Li C., Li C. (2022). Effect of THz spectra of L-Arginine molecules by the combination of water molecules. iScience.

[60] Hou L., Shi W., Dong C., Yang L., Wang Y., Wang H., Hang Y., Xue F. (2021). Probing trace lactose from aqueous solutions by terahertz time-domain spectroscopy. Spectrochim. Acta. A Mol. Biomol. Spectrosc..

[61] Shi W., Wang Y., Hou L., Ma C., Yang L., Dong C., Wang Z., Wang H., Guo J., Xu S. (2021). Detection of living cervical cancer cells by transient terahertz spectroscopy. J. Biophotonics.

[62] Kim S., Chen J., Cheng T., Gindulyte A., He J., He S., Li Q., Shoemaker B.A., Thiessen P.A., Yu B. (2021). PubChem in 2021: New data content and improved web interfaces. Nucleic Acids Res..

[63] Stephens P.J., Devlin F.J., Chabalowski C.F., Frisch M.J. (1994). Ab Initio calculation of vibrational absorption and circular dichroism spectra using density functional force fields. J. Phys. Chem..

[64] Ángyán J.G., Gerber I.C., Savin A., Toulouse J. (2005). van der Waals forces in density functional theory: Perturbational long-range electron-interaction corrections. Phys. Rev. A.

[65] Marenich A.V., Cramer C.J., Truhlar D.G. (2009). Universal solvation model based on solute electron density and on a continuum model of the solvent defined by the bulk dielectric constant and atomic surface tensions. J. Phys. Chem. B.

[66] Lu T., Chen Q. (2021). Interaction region indicator: A simple real space function clearly revealing both chemical bonds and weak interactions. Chem. Methods.

[67] Lu T., Chen F. (2012). Multiwfn: A multifunctional wavefunction analyzer. J. Comput. Chem..

